# Progressive Global Ataxia With Sensory Changes as a Paraneoplastic Syndrome in a Patient With Chromophobe Renal Cell Carcinoma

**DOI:** 10.7759/cureus.24913

**Published:** 2022-05-11

**Authors:** Mustafa M Basree, Raquel Rudy, Cristina Romaniello, Daniel E Smith, Elizabeth Kander

**Affiliations:** 1 Internal Medicine, OhioHealth Riverside Methodist Hospital, Columbus, USA; 2 Internal Medicine, University of Pikeville - Kentucky College of Osteopathic Medicine, Pikeville, USA; 3 Neurology, OhioHealth Riverside Methodist Hospital, Columbus, USA; 4 Hematology/Oncology, Columbus Oncology Associates, Columbus, USA

**Keywords:** multidisciplinary cancer care, paraneoplastic neurological syndromes, renal cell carcinoma (rcc), global ataxia, chromophobe renal carcinoma

## Abstract

Paraneoplastic syndromes (PNS) are rare and can be challenging to diagnose and treat. The uniqueness of PNS lies in the complexity of presentation, the importance of early diagnosis, and the role of multidisciplinary care in managing those patients to mitigate long-term neurologic complications. We describe a patient with metastatic renal cell carcinoma who presented with a complex constellation of neurological symptoms (progressive global ataxia and sensory changes) that did not resolve following nephrectomy. While complete resolution of symptoms was not achieved, he did have stabilization of his neurologic decline with the initiation of cancer-directed therapies.

## Introduction

Paraneoplastic syndromes (PNS) are associated with approximately 10-40% of renal cell carcinoma (RCC) cases, of which, hypercalcemia is the most commonly occurring (in 13-20% of patients) [1,2]. Neurological PNS are rare in RCC. Previous reports have described motor neuron disease, demyelinating polyneuropathies, and myopathies as likely manifestations of paraneoplastic disease in RCC [3]. Typically, neurologic symptoms present prior to the diagnosis of an underlying malignancy and, therefore, are important to recognize early.

## Case presentation

**Figure 1:**
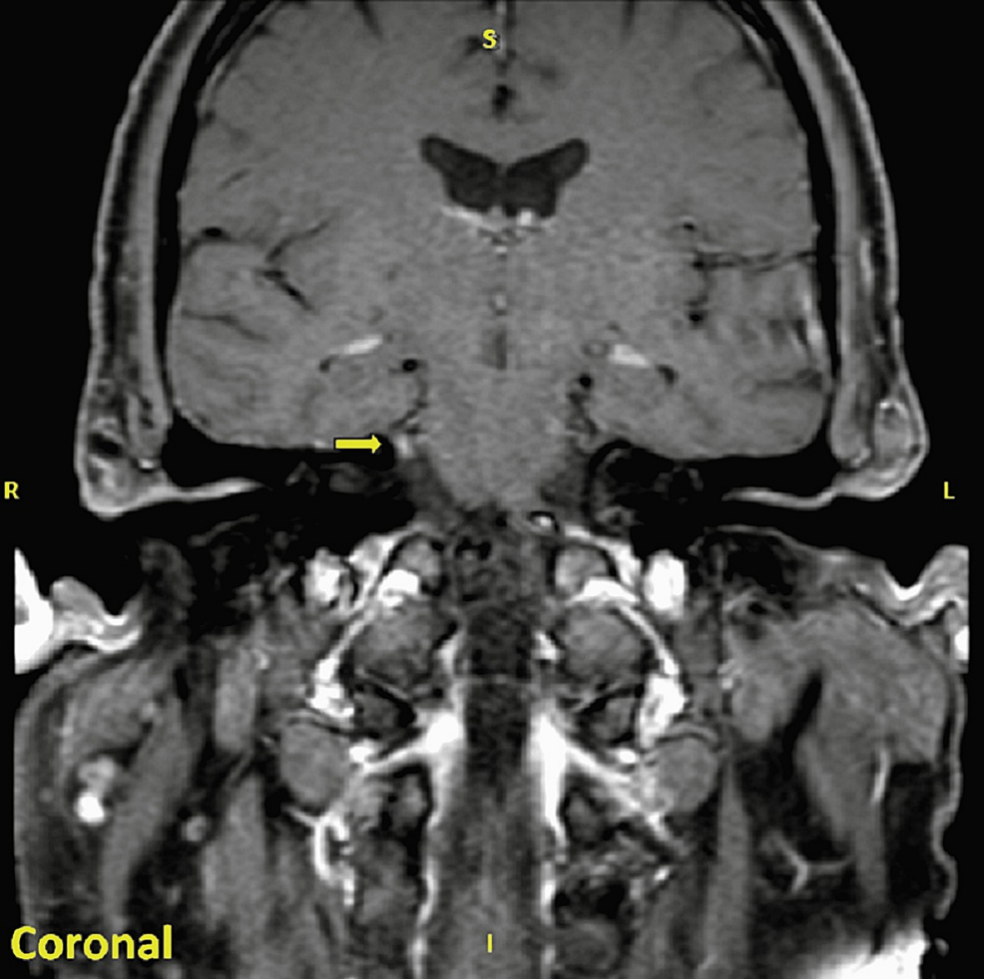
Brain MRI with IV Contrast, Trigeminal Nerve Enhancement T1 thin slice section of brain MRI with IV gadoterate meglumine showing nonspecific enhancement involving the cisternal segment of the right trigeminal nerve extending into Meckel's cave. MRI: magnetic resonance imaging.

A 66-year-old male presented with severe fatigue, binocular horizontal diplopia with lateral ophthalmoplegia, bilateral upper and lower extremity ataxia without dysmetria, and mild subjective decrease in left-sided fine touch sensation. Past surgical history is significant for a recent left rotator cuff repair surgery one month prior to presentation. Brain magnetic resonance imaging (MRI) with intravenous gadoterate meglumine contrast revealed enhancement of the right trigeminal nerve (Figure 1). Lumbar puncture was significant for elevated cerebrospinal fluid (CSF) protein of 98 mg/dL (normal 15-60 mg/dL) and a mild lymphocytic (63%) pleocytosis with 10 white blood cells/uL (normal 0-5 cells/uL). CSF cytology was negative. Infectious workup including serum HIV1/HIV2 antibodies, Borrelia burgdorferi IgM and IgG enzyme-linked immunosorbent assay, and serologic rapid plasma reagin (RPR) was negative. Autoimmune workup including acetylcholine receptor antibodies and anti-muscle-specific kinase was negative. Serum was negative for GQ1b ganglioside antibody. CSF autoantibody testing was positive for low-titer calcium channel binding antibody (P/Q type at a titer of 0.04 nmol/L) of unclear significance and low-level antibody staining for contactin-1, an antibody that has been associated with chronic inflammatory demyelinating polyneuropathy (CIDP), but not typically described in ataxic syndromes. He was treated with intravenous immune globulin IgG (IVIG; 0.4 g/kg daily for five days), methylprednisolone (1 g IV daily for five days), and plasma exchange (PLEX) with subjective improvement in diplopia, however, with persistent ataxia and weakness. He was discharged to an inpatient rehabilitation center with physical and occupational therapy.

**Figure 2:**
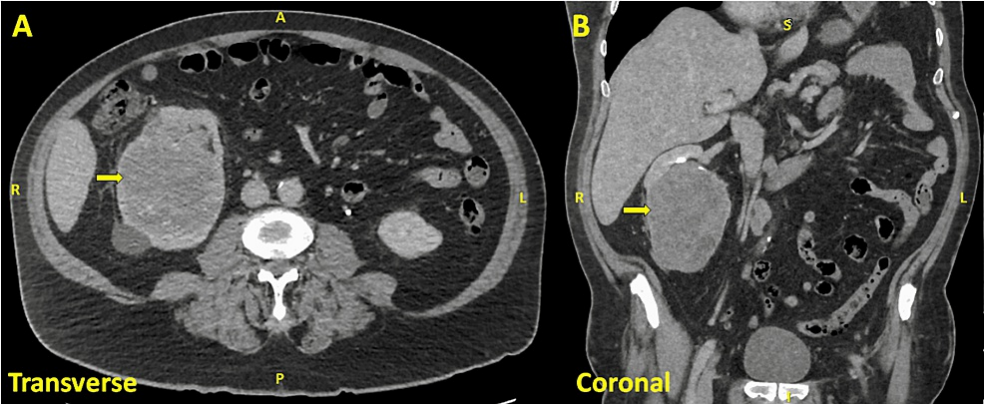
CT Urogram There is an enhancing mass in the lower pole of the right kidney, potentially arising from the renal pelvis, in transverse (A) and coronal (B) planes measuring 9.3 x 10.8 x 11.3 cm (arrows). There are linear areas of hyperdensity within the mass that could reflect calcifications or hemorrhage. The lesion appears to be arising from the renal pelvis. Enlarged retroperitoneal lymph nodes are present in the portacaval region.

He was readmitted to the hospital roughly 1-2 months later because of hematuria and persistent neurological symptoms. Computerized tomography (CT) urogram showed a large right renal mass with retroperitoneal lymphadenopathy (Figure 2A-2B). Right radical nephrectomy revealed a unifocal roughly 11-cm necrotic mass with negative margins. There was evidence of tumor extension into the renal vein and pelvicalyceal system. Three out of three regional lymph nodes showed evidence of malignancy with the largest metastatic deposit measuring 4.5 cm. Immunohistochemistry showed the tumor cells expressed CK7 and CD117 and were negative for carbonic anhydrase IX and vimentin, consistent with chromophobe RCC. There was also a focal sarcomatoid change comprising <5% of the tumor. Over the next few months, despite nephrectomy, he experienced worsening ataxia, progressive weakness now with areflexia involving bilateral upper and lower extremities, decreased fine touch sensation in bilateral distal upper extremities, ophthalmoplegia in all directions, and mild-to-moderate ataxic dysarthria. Of note, electromyography (EMG) of left upper and lower extremities at the time did not show evidence of diffuse neuropathy, myopathy, neuromuscular junction pathology, radiculopathy, or plexopathy. He continued physical and occupational therapy, with the addition of speech therapy.

Treatment was initiated with sunitinib (50 mg by mouth daily for two weeks, then one week off), a multikinase inhibitor, but was poorly tolerated due to fevers, nausea, and diarrhea. Neurological symptoms continued to progress with new bilateral upper and lower extremity dysmetria and dysdiadochokinesia, right upper extremity drift, right facial weakness, occasional dysphagia, and ~50% dysgeusia. Repeat EMG was unchanged from baseline.

Given poor tolerance and interval disease progression, his therapy was changed to cabozantinib (60 mg once daily initially, subsequently reduced to 40 mg then 20 mg due to poor tolerability), another tyrosine kinase inhibitor. He experienced grade 2-3 diarrhea, requiring multiple treatment delays and dose reductions. Given progressive lower extremity weakness and ataxia necessitating a wheelchair for mobility, a brain MRI with intravenous gadoterate meglumine was obtained which demonstrated resolution of prior trigeminal nerve enhancement, but new T2 changes in the left medulla and right dentate nucleus. At this time, he had developed new mild action and postural tremor in both hands. He had a wide-base gait with significant ataxia and motion disequilibrium. He was treated with rituximab (loading dose of 500 mg every two weeks for two doses, followed by a maintenance dose of 1000 mg every six months), an anti-CD20 chimeric antibody. He was able to stand up from wheel chair, balance himself, and take a few steps. This, in conjunction with other therapy, resulted in a subjective sense of feeling well. He completed a two-dose Coronavirus Disease 2019 (COVID-19) vaccination series given immunosuppression while on anti-CD20 therapy.

Several months after initiating rituximab, there were no new changes on exam or brain imaging. He remained on cabozantinib and was able to ambulate 20-30 feet with a walker, but remained wheelchair-dependent for longer distances. The remainder of his symptoms were unchanged until, unfortunately, he contracted COVID-19. He passed away from respiratory failure with severe acute respiratory distress syndrome and bilateral pneumothorax secondary to COVID-19 pneumonia. He received a total of two doses of maintenance rituximab with the last dose roughly two months prior to contracting the virus. The interval between the last vaccination and infection was seven months.

## Discussion

Approximately 10-40% of patients with RCC present with symptoms due to a PNS [2]. PNS symptoms are largely constitutional (i.e., fever), endocrine (i.e., hypercalcemia), or nonendocrine abnormalities (i.e., anemia), with neurologic symptoms (i.e., sensory or motor deficits) being very rare [2]. Paraneoplastic neurological disorders (PND) are rare in any type of malignancy, occurring in less than 1% of cancers. When they do occur, PNDs are most commonly associated with small cell lung cancer, gynecological cancers, breast cancers, and lymphoma [4]. Though rare, there have been reports of paraneoplastic syndromes in RCC presenting as Guillain-Barre Syndrome (GBS) [5], cerebellar ataxia [6], progressive weakness, urinary retention, complex peripheral nervous system syndrome [3], demyelinating peripheral neuropathy [7], Parkinsonism [8], and motor neuron disease resembling amyotrophic lateral sclerosis (Table 1) [9]. The majority of those PNS resolve, or significantly improve, after undergoing definitive management of underlying RCC.

**Table 1:** Published Literature for Neurologic Paraneoplastic Syndromes in Patients with Renal Cell Carcinoma ALS = Amyotrophic Lateral Sclerosis; GBS = Guillain-Barre Syndrome; RCC = Renal Cell Carcinoma; TKI = Tyrosine Kinase Inhibitor; IVIG = Intravenous Immunoglobulin.

Authors; Reference	Year	Article Type	Age, Sex	Neurological Manifestation	Cancer Type/Histology	Treatment	Symptoms Resolved?
Zakrocka et al. [5]	2020	Case report	47, M	GBS	Kidney transplant recipient with allograft RCC	Graftectomy	Yes
Johnson et al. [6]	2008	Case report	53, M	Cerebellar Ataxia	RCC	Nephrectomy	Yes
Yang et al. [3]	2017	Case report	61, F	Progressive weakness, sensory changes, urinary retention, complex peripheral nervous system syndrome	RCC	Nephrectomy	Yes
Nishioka et al. [7]	2017	Case report	50, M	Demyelinating peripheral neuropathy	Clear cell RCC	Nephrectomy, IVIG	Improved
Ali et al. [8]	2017	Case report	74, M	Parkinson-like neurologic syndrome	Advanced clear cell RCC	TKI and consolidative nephrectomy	Yes
Turk et al. [9]	2009	Case report	59, M	Motor neuron disease resembling ALS	RCC	Nephrectomy	Yes

Here we describe a patient with metastatic renal cell carcinoma who presented with progressive global ataxia with sensory changes, a unique neurologic paraneoplastic syndrome not previously described in the literature. Initial hypoesthesia may be confounded by his recent surgery as unilateral presentation is not typically seen in paraneoplastic syndromes; however, this quickly progressed to involve bilateral upper extremities with both sensory and strength deficits. It is unclear if the low-level contactin-1 was significant in his case. Due to a lack of previous reports in managing such complex constellation of neurological symptoms, diagnosis and subsequent management proved to be challenging, especially in the context of persistent nodal disease following nephrectomy. The initial diagnosis was presumed to be Miller Fisher syndrome (a variant of GBS), although GQ1B antibody was negative. PNS became the most likely diagnosis after the discovery of a renal mass on MRI with a lack of response to IVIG, high-dose steroids, and plasmapheresis. Additionally, his progressive neurologic decline despite therapy is not typical of Miller Fischer syndrome which tends to be monophasic.

The presence of onconeural autoantibodies in CSF, while rare, may aid in the diagnosis of an underlying malignancy, although they are not particularly specific for the type of malignancy [10-11]. When autoantibodies are detected, further workup for a paraneoplastic syndrome is needed, including either site-specific imaging and laboratory assessment, or whole-body positron emission tomography (PET), as it can be more sensitive for smaller tumors [12]. Autoantibodies such as anti-contactin-1, which were found in our patient’s CSF, have been reported in a subset of patients with CIDP who exhibited both sensory and motor symptoms [13]. This antibody has not previously been reported in the context of renal cell carcinoma. Other reports have demonstrated an association between contactin-1 and sensory ataxia, likely due to high expression of contactin-1 in dorsal root ganglia neurons [14]. This may also explain a possible connection between contactin-1 and the sensory changes seen in our patient. However, it is also possible that there is another, unknown, antibody responsible for this phenotype, especially in light of his progressive MRI changes.

The approach to managing paraneoplastic syndrome is two-fold: treat the underlying malignancy and minimize neurologic decline. Our patient received definitive management of RCC with surgical resection and systemic therapy [15]. The second goal of minimizing neurologic decline can be complex and requires multiple therapies. It is believed that many of the paraneoplastic syndromes are T-cell mediated, and may be less responsive to immunotherapies compared to non-paraneoplastic autoimmune neurological syndromes that may be directed at cell-surface antigens and are more often B-cell mediated. First-line therapy for neurologic PNS includes IVIG, IV corticosteroids, and PLEX. Rituximab and cyclophosphamide are reserved for second-line therapy [16]. By the time second-line agents are deployed, neuronal damage may have become irreversible, limiting the potential for neurological recovery [17]. Though second-line therapy can confer good clinical outcomes in select patients, the goal of second-line therapy in most patients is stabilization, rather than significant improvement, of neurological symptoms [17]. Treatment of neurological symptoms generally has a positive impact on the prognosis of malignancy [18]. Treatment interruptions, such as those due to intolerance, socioeconomic factors, among others, may impede neurologic recovery.

There is limited data to guide the treatment of patients with locally advanced and/or metastatic non-clear cell RCC (nccRCC) as a majority of studies are limited to those with clear cell RCC [19]. In 2017, a meta-analysis of four randomized trials showed that tyrosine kinase inhibitors (TKIs) offer a significant improvement in progression-free survival (PFS) as compared to mTOR inhibitors, although this did not translate into overall survival (OS) benefit [19-20]. In addition, a multi-center retrospective cohort study of patients with metastatic nccRCC found that cabozantinib had significant anti-tumor activity [21]. Sunitinib and cabozantinib are TKIs that inhibit multiple receptors including vascular endothelial growth factors (VEGF), glial cell-line derived neurotrophic factor receptor (RET), among others resulting in the inhibition of tumor angiogenesis and growth [19-21]. Both agents have demonstrated significant PFS as first- and second-line agents, respectively, for patients with nccRCC [19]. Major side effects of TKIs include hypertension, hand-foot syndrome, fatigue, diarrhea, and thromboembolic events [19,22]. To our knowledge, those agents have not been evaluated in the context of PNS. While there is a role for immune checkpoint inhibitors (i.e., pembrolizumab or nivolumab) in this patient population, they were deferred initially due to concern for GBS, and later due to the constellation and rapid progression of his neurological symptoms. Our patient did not experience complete resolution of his symptoms, potentially due to residual nodal disease and intolerance to therapy. He did, however, have stabilization of his neurologic decline with the initiation of rituximab and cabozantinib. Rituximab is an anti-CD20 B-cell antibody that suppresses humoral immunity [23]. To our knowledge, there are no large, randomized trials evaluating its role in PNS and the majority of literature for use in this context comes from observational studies. Due to its suppressive effect on the immune system, rituximab can cause hepatitis B reactivation, hypogammaglobulinemia, infusion reactions, and progressive multifocal leukoencephalopathy, among others [24]. In the era of COVID-19, it is not inconceivable that anti-CD20 therapy, such as rituximab, may worsen disease course and prognosis, especially given lower vaccine immunogenicity [25]. Those patients could benefit from additional vaccine doses.

Treatment of paraneoplastic syndrome in the context of malignancy has many challenges due to the complexity of the presenting symptoms, as well as, managing treatment of the cancer itself and the paraneoplastic syndrome. For these reasons, multidisciplinary care involving at least an oncologist, neurologist, and allied health professionals (i.e., physical and occupational therapy) is crucial for early diagnosis and for designing appropriate treatment programs tailored to the patient’s specific therapeutic need and clinical course.

## Conclusions

In patients with progressive neurological symptoms without an identifiable cause, it is critical to consider paraneoplastic syndrome. As with our patient, neurological symptoms can precede the diagnosis of malignancy. Although not previously described, the presence of contactin-1 in serum or CSF should prompt further investigation. Early identification and intervention have the potential to significantly improve prognosis in these patients through the arrest of neurologic decline. In patients with significant neurological burden, the goal of management becomes stabilization of symptoms and rehabilitation, which relies on multidisciplinary care involving neurology, oncology, and allied health professionals.

## References

[1] Sacco E, Pinto F, Sasso F, Racioppi M, Gulino G, Volpe A, Bassi P (2009). Paraneoplastic syndromes in patients with urological malignancies. Urol Int.

[2] Palapattu GS, Kristo B, Rajfer J (2002). Paraneoplastic syndromes in urologic malignancy: the many faces of renal cell carcinoma. Rev Urol.

[3] Yang I, Jaros J, Bega D (2017). Paraneoplastic peripheral nervous system manifestations of renal cell carcinoma: a case report and review of the literature. Case Rep Neurol.

[4] Darnell RB, Posner JB (2006). Paraneoplastic syndromes affecting the nervous system. Semin Oncol.

[5] Zakrocka I, Baranowicz-Gąszczyk I, Korolczuk A, Załuska W (2020). Guillain-Barre syndrome: a typical paraneoplastic syndrome in a kidney transplant recipient with allograft renal cell carcinoma: a case report and review of the literature. BMC Nephrol.

[6] Johnson V, Friedman N, Haller NA, Hagel C (2008). Immune mediated neurologic dysfunction as a paraneoplastic syndrome in renal cell carcinoma. J Neurooncol.

[7] Nishioka K, Fujimaki M, Kanai K (2017). Demyelinating peripheral neuropathy due to renal cell carcinoma. Intern Med.

[8] Ali N, Kutikov A, Geynisman DM (2017). Resolution of a debilitating paraneoplastic Parkinson-like neurological syndrome following tyrosine inhibitor therapy and consolidative nephrectomy in a patient with advanced clear cell renal cell carcinoma. Urol Case Rep.

[9] Turk HM, Ozet A, Kuzhan O, Komurcu F, Arpaci F, Ozturk B, Ataergin S (2009). Paraneoplastic motor neuron disease resembling amyotrophic lateral sclerosis in a patient with renal cell carcinoma. Med Princ Pract.

[10] Greenlee JE (2004). Recommended diagnostic criteria for paraneoplastic neurological syndromes. J Neurol Neurosurg Psychiatry.

[11] Fu P, He L, Tang N, Nie Q, Li Z (2021). A single center retrospective study of paraneoplastic neurological syndromes with positive onconeural antibodies. J Clin Neurosci.

[12] Rees JH (2004). Paraneoplastic syndromes: when to suspect, how to confirm, and how to manage. J Neurol Neurosurg Psychiatry.

[13] Cortese A, Lombardi R, Briani C (2020). Antibodies to neurofascin, contactin-1, and contactin-associated protein 1 in CIDP: clinical relevance of IgG isotype. Neurol Neuroimmunol Neuroinflamm.

[14] Miura Y, Devaux JJ, Fukami Y, Manso C, Belghazi M, Wong AH, Yuki N (2015). Contactin 1 IgG4 associates to chronic inflammatory demyelinating polyneuropathy with sensory ataxia. Brain.

[15] Khurana A, Robila V, Massey HD, Paul AK (2020). Paraneoplastic glomerulonephropathy associated with renal cell carcinoma. JCO Oncol Pract.

[16] Devine MF, Kothapalli N, Elkhooly M, Dubey D (2021). Paraneoplastic neurological syndromes: clinical presentations and management. Ther Adv Neurol Disord.

[17] Galli J, Greenlee J (2020). Paraneoplastic diseases of the central nervous system. F1000Res.

[18] Greenlee JE (2010). Treatment of paraneoplastic neurologic disorders. Curr Treat Options Neurol.

[19] (2022). Kidney cancer. https://www.nccn.org/professionals/physician_gls/pdf/kidney.pdf.

[20] Ciccarese C, Iacovelli R, Brunelli M (2017). Addressing the best treatment for non-clear cell renal cell carcinoma: a meta-analysis of randomised clinical trials comparing VEGFR-TKis versus mTORi-targeted therapies. Eur J Cancer.

[21] Martínez Chanzá N, Xie W, Asim Bilen M (2019). Cabozantinib in advanced non-clear-cell renal cell carcinoma: a multicentre, retrospective, cohort study. Lancet Oncol.

[22] Pal SK, Tangen C, Thompson IM Jr (2021). A comparison of sunitinib with cabozantinib, crizotinib, and savolitinib for treatment of advanced papillary renal cell carcinoma: a randomised, open-label, phase 2 trial. Lancet.

[23] Salles G, Barrett M, Foà R (2017). Rituximab in B-cell hematologic malignancies: a review of 20 years of clinical experience. Adv Ther.

[24] Greenlee JE, Carlson NG, Abbatemarco JR, Herdlevær I, Clardy SL, Vedeler CA (2021). Paraneoplastic and other autoimmune encephalitides: antineuronal antibodies, T lymphocytes, and questions of pathogenesis. Front Neurol.

[25] Kroon FP, Najm A, Alunno A, Schoones JW, Landewé RB, Machado PM, Navarro-Compán V (2022). Risk and prognosis of SARS-CoV-2 infection and vaccination against SARS-CoV-2 in rheumatic and musculoskeletal diseases: a systematic literature review to inform EULAR recommendations. Ann Rheum Dis.

